# Transfusion Challenges in Patients with Hematological Malignancies in Sub-Saharan Africa: A Prospective Observational Study from the Uganda Cancer Institute

**DOI:** 10.1038/s41598-020-59773-y

**Published:** 2020-02-18

**Authors:** Henry Ddungu, Elizabeth M. Krantz, Isaac Kajja, Sandra Naluzze, Hanifah Nabbanja, Flavia Nalubwama, Warren Phipps, Jackson Orem, Anna Wald, Noah Kiwanuka

**Affiliations:** 1 Uganda Cancer Institute, Kampala, Uganda; 2 Vaccines and Infectious Diseases Division, Fred Hutchinson Cancer Research Center, Seattle, Washington USA; 3 School of Medicine, College of Health Sciences, Makerere University, Kampala, Uganda; 4 School of Public Health, College of Health Sciences, Makerere University, Kampala, Uganda; 5 Department of Medicine, University of Washington, Seattle, WA USA; 6 Department of Laboratory Medicine, University of Washington, Seattle, WA USA; 7 Department of Epidemiology, University of Washington, Seattle, WA USA

**Keywords:** Health services, Haematological cancer

## Abstract

Blood transfusion is fundamental in managing hematologic malignancies. We sought to evaluate the need and availability of blood products for patients with hematological malignancies at Uganda Cancer Institute. We prospectively studied the demand and supply of blood for patients with thrombocytopenia (platelet count ≤50 × 10^9^/L), anemia (hemoglobin ≤10 g/dL), and bleeding (WHO grade ≥2). We used Poisson generalized estimating equation regression models for longitudinal binary outcomes. Among 91 patients, the median age was 26 years (IQR, 11–47). Thrombocytopenia occurred on ≥1 day in 58% of patients and on 49% of hospital days. Platelets were transfused to 39% of patients. The mean number of platelet units requested per day was 16.2 (range 0–30); 5.1 (range 0–15) were received. Anemia occurred on ≥1 day in 90% of patients; on 78% of days; and 68% received at least one blood transfusion. The mean number of blood units requested was 36.3 (range 8–57) units per day; 14 (range 0–30) were received. Bleeding occurred on ≥1 day in 19% of patients on 8% of hospital days. Thrombocytopenia and anemia were common, but product availability was substantially below that requested. We recommend increased blood collection and adherence to strict transfusion triggers as strategies to improve blood availability.

## Introduction

Cancer therapy is expected to grow substantially in sub-Saharan Africa (SSA) throughout the next decades with up to 1.28 million new cancer cases, 970,000 cancer deaths, and a near doubling of leukemia and lymphoma cases in SSA by 2030^1^. UN Resolution 67/81 urged governments to promote universal health coverage (UHC), where all individuals and communities have access to quality services without financial hardship, as an important element on the international development agenda^2^. Achieving UHC, including access to quality essential health care services and access to safe, effective, quality, and affordable essential medicines, is target 3.8 of the Sustainable Development Goals (SDG)^3^. Indeed, blood and blood components have been added to the WHO Model List of Essential Medicines^4^ and should be available at all times in adequate amounts to fulfill healthcare needs of the population. Most countries in SSA lack centralized national systems for blood collection and provide insufficient transfusion support to meet patient needs. The annual median blood donation rate in low-income countries is 4.6 donations per 1,000 population compared with 32.1 per 1000 population in high-income countries^5^.

Despite the projections of increased cancer care in SSA, there is very little available data on current transfusion support for patients undergoing treatment for hematologic malignancies in SSA. In 2013 the total number of whole blood donations for Uganda was 202,939 units^6^ and for a country with a population of 34 million^7^, the estimated need was 340,000 units for the year (based on a minimum collection aim of 10/1000 population) resulting in a deficit of 40%. Production capacity for platelet transfusion is even lower^8,9^, which is especially relevant for the support of patients with hematologic malignancies undergoing myeloablative chemotherapy.

The Uganda Blood Transfusion Service (UBTS), is the national blood service responsible for providing sufficient and safe blood for the entire country. UBTS is a semi-autonomous department in the Ministry of Health with headquarters in Kampala and operated at 6 regional blood banks each with about 8 staff; 10 blood collection and distribution centers each with 2 staff; and 25 mobile collection teams each composed of 10 members. Blood units from collection centers are delivered to the regional blood banks where blood components i.e. red cell concentrates, platelet concentrates, fresh frozen plasma, and cryoprecipitate are prepared. UBTS collects approximately 320 whole blood units per day of which about 40% are collected as quadruple bags for separation for red blood cells, plasma and platelets; and 60% as single blood bags for collection of whole blood. Of the units collected in quadruple bags, only 40% are used to prepare platelets and plasma (since these have to be separated within 8 hours of collection), but all (100%) quadruple bag units are used to make red blood cells. Blood units for platelet preparation are transported at 24 °C and units for red cell concentrate at 10 °C. Platelets are prepared by the platelet rich plasma method and stored between 22 to 24 °C in a platelet agitator. There is no provision for platelet pooling and no apheresis.

The Uganda Cancer Institute (UCI) on the other hand, is a comprehensive cancer referral hospital for Uganda, registering about 4500 new cancer patients annually, including 430 with hematological malignancies. Blood at UCI is obtained from UBTS and the proportion transfused as whole blood is 60% and as red blood cells is 40%. UCI lacks specific transfusion guidelines and there is no transfusion committee and no specific blood transfusion requisition form as doctors write transfusion orders in patients’ charts. As such, transfusions were generally provided liberally and unevenly throughout the institution. The aim of this study was to evaluate the need and availability of blood products for patients with hematological cancers undergoing treatment at the UCI.

## Materials and Methods

We conducted a prospective observational study among patients admitted to UCI with a hematological malignancy from June 15, 2014 to October 15, 2014. Patients were eligible if they were inpatients of any age with a confirmed hematologic cancer. We excluded patients if they did not have a hematological malignancy or if bleeding was not assessed for the duration of the study. Standardized forms were used to abstract demographic and clinical data, including blood counts, transfusions given, and presence of bleeding from patient records. Blood counts were measured using a Sysmex XN-1000™ hematology analyzer, at least twice weekly, although counts could be ordered more frequently at the physician’s request.

Presence of bleeding was assessed by review of the patient’s chart. If a patient was seen by a doctor but bleeding was not documented in the chart notes, we assumed the patient had no bleeding on that day. Bleeding status was treated as missing on days where the patient was not seen by a doctor or there were no chart notes. Days without mention of transfusions were considered to have no transfusions. For purposes of this study, blood refers to whole blood or red blood cell (RBC) packs and platelets refer to 60–80 mL random donor platelet concentrates. We defined anemia as hemoglobin ≤10 g/dL, thrombocytopenia as platelet count ≤50 × 10^9^/L^10^, and clinically significant bleeding as WHO bleeding grade ≥2^11^. There was no trigger for prophylactic platelet or red cell transfusion, but transfusions were given at the discretion of the attending physician. The number of platelets and blood units that UCI requested and received during the study period was abstracted from UBTS records. Participants were followed until they were discharged or died, for a maximum of 30 days. Enrolled patients who were discharged and readmitted during the study period were only included for the initial admission.

The study was approved by the Makerere University School of Medicine Research and Ethics Committee, the Fred Hutchinson Cancer Research Center (FHCRC) IRB, and the Uganda National Council for Science and Technology. All study methods were performed in accordance with the relevant guidelines and regulations; and informed consent was obtained from all participants and/or their legal guardians.

### Statistical analysis

We described the frequencies and assessed correlates of thrombocytopenia and of bleeding (our two main clinical outcomes) using generalized estimating equations (GEE) with Poisson distribution and log link to account for correlation among longitudinal binary outcomes measured in the same participant^12^. Model estimates were presented as relative risks (RR) with 95% confidence intervals (CI). Continuous independent variables were categorized if the relationship with the outcome was not linear. Because some cancer types had very few participants, we grouped acute myeloid leukemia (AML) with myelodysplastic syndrome (MDS); grouped chronic myeloid leukemia (CML) with chronic lymphocytic leukemia (CLL); and Hodgkin’s lymphoma (HL) with Non-Hodgkin’s lymphoma (NHL). We also omitted from the models one patient with a diagnosis other than any of the specified categories.

Multivariable models were constructed using the purposeful variable selection method^13^. Although primary diagnosis was not significant at the 0.10 level, it had a large effect on the estimates of other variables in the model and was retained in the multivariate model. We computed the proportion of patients who died and used total person-days to compute a 30-day mortality rate with 95% confidence intervals (CI). Because patients were censored when they were discharged, censoring may not have been independent of the mortality outcome. Therefore, our 30-day mortality estimates based upon person-days may be higher than actual.

## Results

### Patient characteristics

We enrolled 104 patients who contributed 1409 follow up days. Thirteen patients were excluded from analysis: four did not have a hematological malignancy; eight did not have bleeding assessed for the duration of the study; and one had no days with platelets measured. The remaining 91 patients contributed 1353 follow up days with a median follow up period of 12 (range 1–31) days. Among the 91 patients, 49% were female; the median age was 26 years (interquartile range 11–47 years) (Table 1). Acute lymphoblastic leukemia (33%), non-Hodgkin lymphoma (22%), and acute myeloid leukemia (18%) accounted for most malignancies; type of malignancy varied by age (Supplemental Table S1). Only 3% were known HIV seropositive, whereas 28% had an unknown HIV status. Most patients (70%) were either currently receiving chemotherapy or had previously received chemotherapy.

**Table 1:** Demographic and clinical characteristics of study participants.

Characteristic	N = 91
**Demographics:**	
Age, median (range) yrs.	26 (2–87)
Female, n (%)	45 (49.45)
**Nationality**, n (%)	
Uganda	88 (96.7)
Rwanda	2 (2.2)
Other	1 (1.1)
**Region of birth**, n (%)	
Central	40 (44.4)
Eastern	13 (14.4)
Western	25 (27.8)
Northern	11 (12.2)
Not in Uganda	1 (1.1)
**Clinical Characteristics:**	
**Primary Diagnosis**, **n (%)**	
Acute lymphocytic leukemia	30 (33.0)
Acute myeloid leukemia	16 (17.6)
Chronic myeloid leukemia	3 (3.3)
Chronic lymphocytic leukemia	6 (6.6)
Hodgkin lymphoma	5 (5.5)
Non-Hodgkin lymphoma	20 (22.0)
Multiple myeloma	9 (9.9)
Myelodysplastic syndrome	1 (1.1)
Other	1 (1.1)
**HIV Status, n (%)**:	
Negative	63 (69.2)
Positive	3 (3.3)
Unknown	25 (27.5)
**Medication (current or past), n (%)**:	
Chemotherapy	63 (70)
Nonsteroidal anti-inflammatory drugs (NSAIDs)	16 (18.2)
**Laboratory studies**, median (range)	
Hemoglobin (g/dL)	7.9 (4.1–13.9)
Platelets (×10^9^/L)	63.3 (3.9–611)
WBC (×10^9^/L)	6.3 (0.47–426)

### Anemia and blood transfusions

Patients had blood counts measured on a median of 4 days (range 1–13 days). A hemoglobin of ≤10 g/dL was recorded on at least one day in 90% of patients and on 316 (78%) of 403 total days with blood counts measured (Table 2). At least one blood transfusion was given to 62 (68%) patients and on 193 (15%) of 1257 days of observation. The median pre-transfusion hemoglobin was 6.6 g/dL (range 3.2–18.0 g/dL). Most blood transfusions (79%) were given for anemia only without evidence of bleeding. Patients 0 to 15 years had the lowest median percentage of days with blood transfusion (6.9%) while those 31 to 45 years old had the highest median percentage of days with blood transfusions (22.2%). Blood transfusion was less common for patients with ALL (median 9.0% of days) than for patients with other hematologic malignancies (median 16.7%). For patients with CML/CLL and HL/NHL the distribution of percentage of days with blood transfusions was bimodal, with some patients receiving no blood transfusions and others receiving numerous transfusions (data not shown).

**Table 2:** Frequency of anemia, blood transfusions, thrombocytopenia, platelet transfusions and bleeding among patients during the study period.

Outcome	Patients (n = 91)
**Anemia (Hb** ≤**10 g/dL)**	
Number of patients with anemia on at least 1 day (%)	82 (90.1%)
Number of days with anemia/Total days with Hb measured (%)	316/403 (78.4%)
**Blood transfusion**	
Number of patients with at least one blood transfusion (%)	62 (68.1%)
Number of days with blood transfusion/Total days with transfusion assessed (%)	193/1257 (15.4%)
**Reason for transfusion, n (%):**	
Acute bleeding only	15 (7.8%)
Anemia only	152 (78.8%)
Acute bleeding and anemia	14 (7.2%)
Other	12 (6.2%)
Median pre-transfusion hemoglobin (g/dL), (range)	6.6 (3.2–18.0)
**Number of blood units given per blood transfusion (%)**	
One unit	188 (98.9%)
Two units	2 (1.1%)
**Thrombocytopenia (platelet count of** ≤**50** × **10**^**9**^**/L)**	
Number of patients with thrombocytopenia on at least 1 day (%)	53 (58.2%)
Number of days with thrombocytopenia/Total days with platelets measured (%)	196/403 (48.6%)
**Platelet Transfusions:**	
Number of patients with at least one platelet transfusion (%)	35 (38.5%)
Number of days with platelet transfusion/Total days with transfusion assessed (%)	90/1257 (7.2%)
**Reason for transfusion, n (%):**	
Prophylactic for low platelets only	56 (62.2%)
Therapeutic for active bleeding only	29 (32.2%)
Prophylactic for low platelets and therapeutic for active bleeding	3 (3.3%)
Pre-invasive procedure	0
Other	2 (2.2%)
Median pre-transfusion platelet count (×10^9^/L), (range)	10.7 (0.1–72)
**Number of platelet units given per platelet transfusion (%):**	
1	10 (12.2%)
2	50 (61.0%)
3	14 (17.1%)
≥4	8 (9.7%)
**Bleeding**	
Number of patients with WHO grade ≥2 bleeding on at least 1 day (%)	17 (18.7%)
Number of days with WHO grade ≥2 bleeding/Total days with bleeding assessed (%)	73/893 (8.2%)

**Figure 1:**
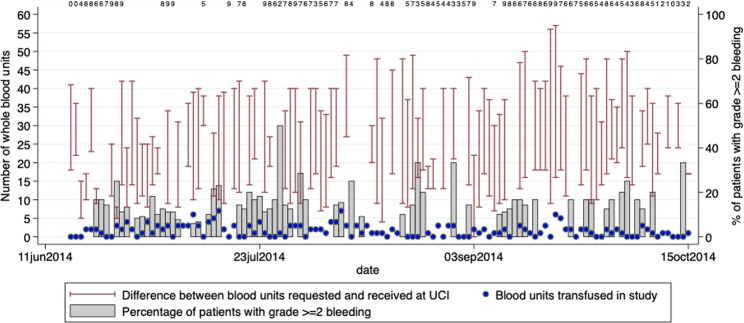
Daily availability of blood units. Red bars indicate difference between number of units requested (top of the bar) and number received (bottom of the bar). Blue dots represent number of units transfused. Grey bars represent percentage of patients with grade ≥2 bleeding on that day. The numbers listed at the top of the graph show the number of patients with bleeding assessed for each day with less than 10 patients with bleeding assessed.

The total number of blood units requested and received by UCI was available from UBTS records for 90 days of the 4-month study period. During this time, the mean daily number of blood units requested was 36.3 (range 8–57) as compared with a mean of 14 (range 0–30) units that were actually received. Accordingly, the median percentage of requested blood units that were received per day was 36% (range 0–100%). Overall, the total number of blood units transfused to participants during the study period was 193 units and almost all transfusions (99%) were one unit per transfusion episode (Fig. 1).

### Thrombocytopenia and platelet transfusions

Thrombocytopenia occurred on at least one day in 58% of patients and on 196 of 403 (49%) days (Table 2). Platelet transfusions were given to 35 (39%) patients and on 90 of 1257 (7%) days, with a median pre-transfusion platelet count of 10.7 × 10^9^/L (range 0.1–72 × 10^9^/L). The primary indication for platelet transfusion was prophylaxis for low platelets (62% of transfusions), active bleeding without thrombocytopenia (32%), and active bleeding with thrombocytopenia (3%). Platelet transfusions were more common among those 0–30 years (median of 3.6% of days) than patients older than 30 years, who rarely received platelet transfusions (median of 0% of days). Patients with ALL and AML were more likely to receive multiple platelet transfusions (median of 4.5% of days) than patients with other diagnoses (median of 0% of days).

**Figure 2:**
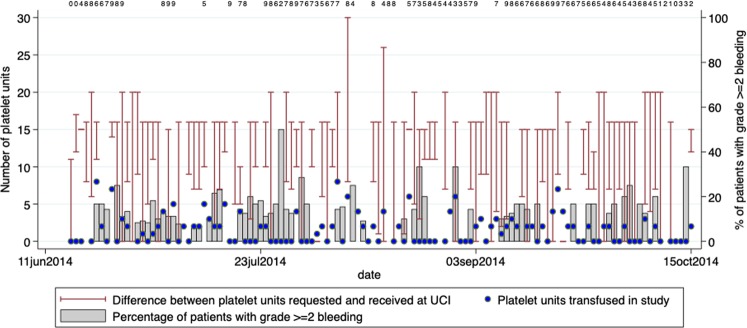
Daily availability of platelet units. Red bars indicate the difference between the number of units requested (top of the bar) and the number received (bottom of the bar). The blue dots represent the number of units transfused to patients. Grey bars represent the percentage of patients with WHO grade ≥2 bleeding on that day. The numbers listed at the top of the graph show the number of patients with bleeding assessed for each day with less than 10 patients with bleeding assessed.

During the 90-day period with aggregate data available, the mean daily number of platelet units requested was 16.2 (range 0–30 units) but the mean number of platelet units received was only 5.1 (range 0–15). Thus, a median of 35% (range 0–100%) of requested platelet units was received. On 95% of days with data available on products requested and provided, UBTS was unable to supply more than 75% of the requested number of units. During the study period, the total number of platelet units transfused to study participants was 186 units. Most transfusions (61%) had 2 platelet units given with only 10% of patients receiving 4–6 units (the international standard for an adult patient) per transfusion (Fig. 2).

### Correlates of thrombocytopenia

In unadjusted analysis, the only significant correlate of thrombocytopenia was cancer type: patients with multiple myeloma had a significantly lower likelihood of thrombocytopenia when compared to patients with ALL (unadjusted RR = 0.1; 95% CI 0.01 to 0.6; p = 0.01) (Table 3).

**Table 3:** Risk factors for thrombocytopenia.

Potential Baseline Correlates		Unadjusted RR (95% CI)	*p*-value	Adjusted RR^a^ (95% CI)	*p*-value
Sex	Female	0.7 (0.5–1.1)	0.12	0.6 (0.4–0.9)	0.008
Age categories (years)	<16 (referent)	1.0		1.0	
	16 to 30	1.3 (0.9–2.1)	0.18	1.7 (1.1–2.7)	0.02
	31 to 45	1.0 (0.5–2.2)	0.91	2.0 (1.2–3.4)	0.008
	46 to 60	0.9 (0.3–2.7)	0.87	1.3 (0.7–2.5)	0.45
	>60	0.8 (0.4–1.7)	0.64	1.1 (0.5–2.6)	0.76
Baseline hemoglobin (g/dL)	Per one-unit decrease	1.1 (1.0–1.2)	0.12	1.1 (1.02–1.2)	0.02
Primary diagnosis	ALL (referent)	1.0		1.0	
	AML/MDS	1.3 (0.9–2.0)	0.16	1.0 (0.6–1.5)	0.88
	CML/CLL	1.4 (0.8–2.5)	0.22	1.2 (0.7–2.1)	0.46
	HL/NHL	0.7 (0.4–1.2)	0.17	0.4 (0.2–0.7)	0.001
	Multiple myeloma	0.1 (0.01–0.6)	0.01	0.04 (0.01–0.3)	0.003
Chemotherapy used at or before enrollment		1.4 (0.8–2.5)	0.18	1.7 (1.1–2.8)	0.02
NSAIDs used at or before enrollment		1.3 (0.8–2.1)	0.21	1.5 (1.02–2.2)	0.04

^a^Adjusted for sex, age, baseline hemoglobin, cancer type, chemotherapy and NSAIDs use.

In multivariable analysis, female patients were less likely to have thrombocytopenia relative to males (adj. RR = 0.6, 95% CI; 0.4 to 0.9; p = 0.008). Compared with those younger than 16 years, 16–30-year-old patients (adjusted RR = 1.7, 95% CI 1.1 to 2.7; p = 0.02) and 31–45-year-old patients (adj. RR = 2.0, 95% CI 1.2 to 3.4; p = 0.008) were twice as likely to have thrombocytopenia. Thrombocytopenia and anemia were correlated and each 1 g/dL lower in baseline hemoglobin was associated with a 12% higher likelihood of thrombocytopenia (adjusted RR = 1.12, 95% CI 1.02 to 1.23; p = 0.02). The use of non-steroidal anti-inflammatory drugs (NSAIDs) (adj. RR = 1.5, 95% CI 1.02 to 2.2; p = 0.04) and chemotherapy (adj. RR = 1.7, 95% CI 1.1 to 2.8; p = 0.02) were also associated with increased presence of thrombocytopenia. Patients with lymphoma and those with multiple myeloma were significantly less likely to have thrombocytopenia than patients with ALL. Patients with AML/MDS or CLL/CML had a similar risk of thrombocytopenia as patients with ALL (Table 3).

### Bleeding and mortality

Bleeding was assessed on a median of 7 days per patient (range 1–29 days). Seventeen patients (18.7%) had clinically significant bleeding on at least one day. Among patients with any bleeding, the highest grade of bleeding was grade 2 for 58.7% of patients and grade 1 for 41.4% of patients; no patients had any grade 3 or 4 bleeding. Of the 893 total days with bleeding assessed, 51 days (5.7%) had grade 1 bleeding and 73 (8.2%) days had grade 2 bleeding reported.

In unadjusted analysis, grade 2 bleeding was more common among those 16 years or older than those younger than 16 years (Table 4). Patients aged 16 to 30 years (unadjusted RR = 8.5, 95% CI 1.9–38.7; p = 0.006) and those above 60 years (unadjusted RR 6.2, 95% CI 1.4 to 26.7; p = 0.02) had significantly higher risk of grade 2 bleeding than those aged 0–15 years. Patients who were 31 to 45 and 46 to 60 years old also had an increased likelihood of grade 2 bleeding compared with those 0–15 years old, though these differences were not statistically significant. Compared to patients with baseline platelet counts >30 × 10^9^/L, those with platelet counts ≤10 × 10^9^/L (RR 8.1, 95% CI 2.1 to 30.8; p = 0.002) and >20 to ≤30 × 10^9^/L (RR 5.2, 95% CI 1.4 to 19.7; p = 0.02) had a greater chance of grade 2 bleeding. However, patients with baseline platelet counts >10 to ≤20 × 10^9^/L did not have significantly greater likelihood of grade 2 bleeding when compared to patients with baseline platelet counts >30 × 10^9^/L. Baseline hemoglobin was not associated with risk of bleeding but a diagnosis of CML/CLL was associated with a 12 times higher risk of grade 2 bleeding than a diagnosis of ALL (unadjusted RR 12.3, 95% CI 3.8 to 39.4; p < 0.001). Patients with other diagnoses did not have significantly different bleeding risk than those with ALL. Chemotherapy use was associated with a higher risk of grade 2 bleeding (RR 4.3, 95% CI 1.2 to 15.3; p = 0.03) whereas NSAIDs use was not.

**Table 4:** Risk factors for WHO grade ≥2 bleeding.

Potential Baseline Correlates		Unadjusted RR (95% CI)	*p*-value	Adjusted RR^a^ (95% CI)	*p*-value
Sex	Male	1.0		1.0	
	Female	2.2 (0.5–8.8)	0.28	2.4 (1.0–5.8)	0.06
Age categories (in years)	0 to 15	1.0		1.0	
	16 to 30	8.5 (1.9–38.7)	0.006	5.3 (0.9–29.9)	0.06
	31 to 45	2.3 (0.4–13.7)	0.37	1.0 (0.2–4.8)	0.99
	46 to 60	5.2 (0.8–34.3)	0.09	3.3 (0.7–16.3)	0.15
	>60	6.2 (1.4–26.7)	0.02	3.9 (0.4–37.2)	0.23
Baseline platelet count	≤10 × 10^9^/L	8.1 (2.1–30.8)	0.002	2.6 (0.6–12.0)	0.23
	>10 to ≤20 × 10^9^/L	1.6 (0.3–9.9)	0.60	1.4 (0.2–9.0)	0.73
	>20 to ≤30 × 10^9^/L	5.2 (1.4–19.7)	0.02	7.5 (1.6–34.3)	0.01
	>30 × 10^9^/L	1.0		1.0	
Baseline hemoglobin (g/dL)	>4 to ≤7	1.0			
	>7 to ≤10	1.8 (0.4–7.0)	0.43		
	>10	1.6 (0.3–7.8)	0.53		
Primary diagnosis	ALL	1.0		1.0	
	AML/MDS	2.9 (0.7–13.0)	0.16	1.5 (0.6–3.8)	0.35
	CML/CLL	12.3 (3.8–39.4)	<0.001	2.8 (0.8–9.9)	0.12
	HL/NHL	0.9 (0.2–5.7)	0.94	0.9 (0.2–4.6)	0.93
	Multiple Myeloma	2.4 (0.3–19.3)	0.42	4.6 (0.5–40.0)	0.17
Chemotherapy used at or before enrollment	No	1.0		1.0	
	Yes	4.3 (1.2–15.3)	0.03	2.5 (1.0–6.0)	0.04
NSAIDS used at or before enrollment	No	1.0			
	Yes	1.2 (0.4–4.1)	0.72		

^a^Adjusted for sex, age, baseline platelet count, cancer type, and chemotherapy use.

In multivariable analysis (Table 4), there was a trend toward higher risk of bleeding among female patients (adjusted RR 2.4, 95% CI 1.0 to 5.8; p = 0.06). The pattern of increased risk of bleeding among adults 16 years or older compared to those 0–15 years old remained, though adjusted estimates were attenuated, and individual comparisons were not statistically significant. Compared to patients with baseline platelet counts >30 × 10^9^/L, those with platelet counts >20 to ≤30 × 10^9^/L had greater risk of grade 2 bleeding (adjusted RR 7.5, 95% CI 1.6 to 34.3; p = 0.01). However, patients with platelet counts below 20 × 10^9^/L did not have significantly greater risk of bleeding when compared to patients with platelet counts >30 × 10^9^/L. Use of chemotherapy at baseline remained associated with higher risk of bleeding, though the estimate was attenuated (adjusted RR 2.5, 95% CI 1.0 to 6.0; p = 0.04).

Fourteen (15.4%) patients died during the study period for a 30-day mortality rate of 31.0%, (95% CI 18.4% to 52.4%). Most of these deaths were clinically attributed to sepsis.

## Discussion

In this prospective observational study, we found that anemia and thrombocytopenia were common among patients treated for cancer in Uganda. Most patients received at least one blood transfusion and 39% received platelet transfusion. While the median pre-transfusion hemoglobin was low (6.6 g/dl), there was a wide range of pre-transfusion hemoglobin values. Importantly, the availability of blood products from the national transfusion service was low, with UCI receiving only one third of the blood and platelet units requested each day. The underlying causes of blood shortage include the significantly low whole blood donation rate in Uganda (as it is for many sub-Saharan African countries); inadequate capacity to screen the donated blood; and financial challenges of processing blood into the various components.

The World Health Assembly expressed concern about the global inequality of access to blood products leaving many patients without this needed treatment and urged Member States to ensure achieving self-sufficiency in the supply of safe blood without shortages^14^. Few studies have examined the demand for and availability of blood products in SSA^6^, and we are not aware of any prior prospective study quantifying the challenges of transfusion support for cancer care in SSA. One study at a tertiary hospital in Uganda found a low availability of blood products, especially platelets^15^.

Blood donation in Uganda is based on voluntary non-remunerated blood donors (VNRBD) as recommended^14^ although such a system is a challenge as the number of donations per 1000 population in SSA is far lower than in high-income nations probably contributing to the shortfall between supply and demand that we observed. Our data highlight a high percentage of unmet requests for both blood and platelets, receiving just about a third of the amount requested. We also found that blood and platelet transfusions were given for a wide range of pre-transfusion hemoglobin and platelet counts.

Given the scarcity of blood products, strategies that decrease their use while preventing complications in cancer patients are urgently needed. One such strategy is to omit prophylactic platelet transfusion and only use platelets for bleeding events (therapeutic transfusion). In a study by Wandt *et al*., they found that a therapeutic platelet transfusion strategy can be performed safely and reduces the need for prophylactic transfusions^16^. However, a randomized trial in United Kingdom and Australia (TOPPS) in which patients were randomly assigned to receive, or not to receive prophylactic platelets at a threshold of 10 × 10^9^/L, supported use of prophylactic platelet transfusion for high risk patients^17^. More recently, our group reported that the same threshold is also reasonable in SSA^18^. Improved utilization of blood products may also be achieved by stricter adherence to transfusion trigger guidelines. A study in Malawi found that strict enforcement of a transfusion protocol reduced the percentage of severely anemic children transfused from 44% to 11%, without adverse effects on mortality^19^. Other studies have also supported benefit for adherence to restrictive transfusion guidelines^20,21^. Furthermore, physicians could consider alternatives to transfusion and implement strategies that include pharmacological correction of anemia using erythropoietin (although expensive), oral or intravenous iron (in selected patients), and use of antifibrinolytics (e.g. epsilon aminocaproic acid) to prevent bleeding in patients with thrombocytopenia^22–24^.

In multivariable analysis to refine clinical correlates of transfusion need, thrombocytopenia was more likely among male patients and young adults compared with younger patients. Patients with lymphoma or multiple myeloma had lower likelihood of thrombocytopenia than patients with ALL. This result was expected, as patients with lymphoma or myeloma rarely present with thrombocytopenia unless they have bone marrow involvement with lymphoma or leukemic transformation^25^. As in other studies, grade 2 bleeding was common in our patients, but we did not observe any grade 3 or 4 events although our patients did not have access to brain CT scans and so smaller central nervous system bleeds could have been overlooked. We could not find any studies in SSA that reported the severity of bleeding outcomes among hematological malignancy patients for comparison. However, a study in Bangkok, Thailand, intracranial hemorrhage (grade 4 bleeding) was found in 1.1% of patients; patients with AML had higher risk^26^.

We acknowledge limitations to this study. This was an observational study where we relied on data from patient charts. However, it was prospective, and we sought clarification from the clinical team whenever notes were unclear. Our sample size was modest relative to the number of parameters included in our multivariable models and associations should therefore be interpreted with caution. We may have missed some clinically significant bleeding events though we feel it would be unlikely that documentation of severe bleeding would have been omitted. We used the WHO bleeding scale to assess for presence of bleeding which is limited by dependence on clinician interpretation of patient recall and subjectivity associated with assigning a grade of bleeding making it difficult to define a significant bleeding. We were not able to determine the outcome of patients with an indication for a blood transfusion but were not transfused because of lack of supply.

In conclusion, this is the first study to quantify the demand and supply of transfusion support for patients with hematological cancers in SSA. Thrombocytopenia and anemia were common but significant bleeding resulting from thrombocytopenia was rare. The low availability of blood products, as it is for other low-income countries with a median blood donation rate of 4.6 donations per 1000 people^5^, resulted in fewer transfusions than requested at UCI where blood management principles are non-restrictive. The wide range of pre-transfusion platelet counts, and hemoglobin levels suggests that stricter transfusion guidelines may help focus limited resources to those most in need. Implementation of guidelines with periodic reviews of adherence and increased blood collection may improve utilization of blood products in SSA. Strategies to reduce the use of blood, such as pharmacological measures to reduce bleeding, should be further explored to enable the optimal use of the limited blood supply.

## Supplementary information

Supplementary Table S1.

## Data Availability

The datasets used and/or analyzed during the current study are available from the corresponding author on reasonable request.
