# Supplementary material for: Transfusion Challenges in Patients with Hematological Malignancies in Sub-Saharan Africa: A Prospective Observational Study from the Uganda Cancer Institute
Source: Sci Rep. 2020 Feb 18;10:2825. doi: 10.1038/s41598-020-59773-y (PMC7028934; doi:10.1038/s41598-020-59773-y)
Supplement: Supplementary file 1 — Supplementary Table S1. [file 41598_2020_59773_MOESM1_ESM.docx]

**Transfusion Challenges in Patients with Hematological Malignancies in Sub-Saharan Africa: A Prospective Observational Study from the Uganda Cancer Institute**

Supplementary Table S1

Table 1: Distribution of Age Categories by Primary Diagnosis

| Diagnosis | **Age categories at enrollment (years)** | | | | | **Total** |
| --- | --- | --- | --- | --- | --- | --- |
|  | 0-15 | 16-30 | 31-45 | 46-60 | >60 |  |
| ALL | 23 | 6 | 1 | 0 | 0 | **30** |
| AML/MDS | 4 | 8 | 2 | 1 | 2 | **17** |
| CML/CLL | 0 | 1 | 1 | 3 | 4 | **9** |
| HL/NHL | 2 | 8 | 4 | 4 | 7 | **25** |
| Multiple myeloma | 0 | 1 | 5 | 2 | 1 | **9** |
| **Total** | **29** | **24** | **13** | **10** | **14** | **90** |

Authors: ^*^Henry Ddungu^1,3^, Elizabeth M. Krantz^2^, Isaac Kajja^3^, Sandra Naluzze^1^, Hanifah Nabbanja^1^, Flavia Nalubwama^1^, Warren Phipps^2,5^, Jackson Orem^1^, Anna Wald^2,5,6,7^, Noah Kiwanuka^4^

Author Institutions: ^1^ Uganda Cancer Institute, Kampala, Uganda

^2^Vaccines and Infectious Diseases Division, Fred Hutchinson Cancer Research Center, Seattle, Washington, USA

^3^School of Medicine, College of Health Sciences, Makerere University, Kampala, Uganda.

^4^School of Public Health, College of Health Sciences, Makerere University, Kampala, Uganda.

^5^ Department of Medicine, University of Washington, Seattle, WA, USA

^6^ Department of Laboratory Medicine, University of Washington, Seattle, WA, USA

^7^ Department of Epidemiology, University of Washington, Seattle, WA, USA

^*^Corresponding author address:

Uganda Cancer Institute,

Upper Mulago Hill Road.

PO Box 3935 Kampala, Uganda

Tel: 256 77 242 6806

Email: [hddungu@fredhutch.org](mailto:hddungu@fredhutch.org)
